# Aversive tension in female adolescents with Anorexia Nervosa: a controlled ecological momentary assessment using smartphones

**DOI:** 10.1186/s12888-016-0807-8

**Published:** 2016-04-12

**Authors:** David R. Kolar, Florian Hammerle, Ekkehart Jenetzky, Michael Huss, Arne Bürger

**Affiliations:** Department for Child and Adolescent Psychiatry and Psychotherapy, University Medical Center of the Johannes Gutenberg University, Mainz, Germany; Division of Clinical Epidemiology and Aging Research, German Cancer Research Center, Heidelberg, Germany; Department of Child and Adolescent Psychiatry, Psychosomatics and Psychotherapy, University Hospital of Würzburg, Würzburg, Germany

**Keywords:** Anorexia nervosa, Eating disorder, Adolescence, Aversive tension, Ecological momentary assessment, Smartphones, Emotion regulation

## Abstract

**Background:**

Current models of Anorexia Nervosa (AN) emphasize the role of emotion regulation. Aversive tension, described as a state of intense arousal and negative valence, is considered to be a link between emotional events and disordered eating. Recent research focused only on adult patients, and mainly general emotion regulation traits were studied. However, the momentary occurrence of aversive tension, particularly in adolescents with AN, has not been previously studied.

**Method:**

20 female adolescents with AN in outpatient treatment and 20 healthy adolescents aged 12 to 19 years participated in an ecological momentary assessment using their smartphones. Current states of aversive tension and events were assessed hourly for two consecutive weekdays. Mean and maximum values of aversive tension were compared. Multilevel analyses were computed to test the influence of time and reported events on aversive tension. The effect of reported events on subsequent changes of aversive tension in patients with AN were additionally tested in a multilevel model.

**Results:**

AN patients showed higher mean and maximum levels of aversive tension. In a multilevel model, reported food intake was associated with higher levels of aversive tension in the AN group, whereas reported school or sport-related events were not linked to specific states of aversive tension. After food intake, subsequent increases of aversive tension were diminished and decreases of aversive tension were induced in adolescents with AN.

**Conclusions:**

Aversive tension may play a substantial role in the psychopathology of AN, particular in relation with food intake. Therefore, treatment should consider aversive tension as a possible intervening variable during refeeding. Our findings encourage further research on aversive tension and its link to disordered eating.

**Trial registration:**

German register of clinical trials (DRKS): DRKS00005228 (Date of registration: September 2, 2013).

## Background

In the last decade, emotion regulation deficits in Anorexia Nervosa (AN) have received increased research interest as they were identified as possible risk and maintaining factors for dysfunctional eating behavior [1–6]. A theoretical framework of the influence of emotion regulation on AN is provided by Haynos and Fruzzetti [7], suggesting that emotional vulnerability, emotional events, and internal judgments, lead to emotional dysregulation. A key feature of emotional dysregulation is aversive tension, which was initially reported by patients suffering from borderline personality disorder (BPD) [8]. Aversive tension is described as a current, dysregulated emotional state of intense arousal and negative valence. The emotional state is most often not linked to a specific emotion and therefore generalized, accompanied by a strong urge for termination [8–10]. States of aversive tension are frequently experienced in daily life with a meaningful variation over time even in the general population, but to a lesser degree than in clinical populations [10, 11]. Therefore, aversive tension is a situation-dependent state rather than a personality trait, and differs from related concepts such as negative emotionality, fear of change, and neuroticism, which should remain stable during daily course. In psychiatric research, aversive tension is primarily studied in BPD [8, 12, 13], but is also found in other mental disorders (e.g. anxiety and mood disorders) [10].

According to the model of Haynos and Fruzzetti, disordered eating, such as restriction or purging, serves as a maladaptive strategy for the reduction of aversive tension and emotional dysregulation. In a recent ecological momentary assessment (EMA) study, higher levels of negative affect on day one predicted a greater likelihood of restrictive eating on day two and negative affect decreased significantly after compensatory behavior [14]. Another study did not find any differences in negative affect or tension when comparing high and low restriction days in adults with AN [15]. Thus, Haynos et al. [15] argue that restriction presumably serves as a “downregulation” rather than an avoidance strategy. This implies that restriction regulates recently triggered affective states instead of permanent affective traits. As both studies do not examine the precipitants of negative affect, it remains unclear whether cognitive variables, such as anticipation of negative affect or situational variables (e.g. meal times, demanding or stressful work situations, and interpersonal conflict), result in increased tension or negative affect, and subsequently lead to disordered eating. Identifying the precipitants of aversive tension will therefore contribute to a better understanding of the causal processes leading to disordered eating in individuals with AN.

Regarding emotional events associated with aversive tension in individuals with AN, there is evidence that food and/or body-related events provoke negative affect and emotion dysregulation [16–18]. Furthermore, individuals with AN tend to avoid emotional situations and have a greater repertoire of maladaptive emotion regulation strategies [19]. Because AN is associated with personality traits such as perfectionism and obsessive-compulsiveness [20], school situations requiring performance could be of additional distress, whereas sport is considered to be perceived positively by individuals with AN [21]. However, the role of emotional events such as food intake, school or sport-related events, on aversive tension in daily life remains unclear. In addition, most of the work on emotion regulation is based on adult individuals. Zimmermann and Iwanski [22] identified dramatic age differences of the emotion regulation strategies available with the smallest repertoire in middle adolescence. Hence, aversive tension might be more often experienced by adolescents in general and emotion dysregulation associated with AN may change from adolescence to adulthood.

Aversive tension is of special interest in dialectical behavior therapy (DBT), and DBT is considered a suitable treatment for AN [23–26]. Reducing aversive tension is a core issue of traditional DBT for pervasive emotion dysregulation disorders [27], but its relevance is also discussed in DBT for AN. Recently, Lynch et al. [28] published an alternative DBT manual for eating disorders, proposing that restrictive eating is a consequence of overcontrol and “emotional loneliness”, rather than a regulation strategy for aversive tension. Subsequently, teaching distress tolerance skills is significantly reduced in the radically-open DBT approach proposed by Lynch et al. [28] Although AN restrictive subtype is related to overcontrol personality traits such as perfectionism [20], it is yet unclear if overcontrol automatically assumes lower levels of aversive tension. Therefore, investigating the extent to which aversive tension is important to individuals with AN has direct implications for further development of DBT for AN.

In previous studies, aversive tension was mostly researched with EMA studies [11, 12]. EMA designs show significantly reduced retrospective bias for self-report data [29]. Recent EMA studies on affect regulation in individuals with AN showed good feasibility and reliability of the method in this population [14, 30–32]. Because most adolescents possess smartphones, which can serve as real-life monitoring devices, conducting EMA studies on personal smartphones appears to be a suitable research option to increase data quality and to reduce research burden.

In conclusion, there is significant indication that aversive tension might be an important factor in individuals with AN. With the model of Haynos and Fruzzetti, a theoretical approach to emotion dysregulation in AN exists, but the assumption of experience of aversive tension in individuals with AN has not been verified yet. This seems of particular interest as modern treatments for AN (e.g. DBT) focus on reducing aversive tension without empirical evidence of this construct in this condition. Furthermore, as most of the studies on emotion regulation and AN are based on adult patients, studies investigating elements of emotion dysregulation in adolescents with AN are required.

The aim of this study was to evaluate if emotional events were linked to increased aversive tension, as postulated in Haynos’ and Fruzzetti’s [7] emotion dysregulation model of AN. More specifically, we were interested in determining whether adolescents with AN differed from healthy adolescents with regard to the experience of aversive tension. Additionally, we wanted to examine if specific events were linked to momentary and subsequent levels of aversive tension, both in general and in the case of AN. We therefore hypothesized that adolescents with AN (1) reported higher mean levels and (2) higher maximum levels of aversive tension (main effects). Furthermore, we expected that, compared to healthy controls, report of food intake and school events was associated with higher momentary levels of aversive tension, whereas reported sport-related events were associated with lower levels of aversive tension in adolescents with AN (interaction effect of AN and events). In addition, we expected an increase of aversive tension after food intake and school-related events, whereas sport-related events should decrease aversive tension of individuals with AN in the subsequent measurement interval (main effect of event).

## Methods

### Participants

Participants with completed datasets included 40 female adolescents, 20 of whom were control participants and 20 of whom met the DSM-5 [33] criteria for AN. Participants were included if they were aged between 12 to 19 years, female, and were experienced with smartphones. Exclusion criteria for the patient group were: 1) presumed or confirmed diagnosis of a personality disorder, when suspected during diagnostic assessment by an experienced clinician, 2) BMI percentile < 3 leading to somatic complications, in line with national guidelines for outpatient treatment of eating disorders [34]. Exclusion criteria for the control group were 1) if stating that one received psychiatric treatment or psychotherapy in the last five years or 2) a high symptom burden operationalized with a GSI T-value above 63 in the symptom check list (SCL-90-R) [35]. One enrolled control participant was excluded because of a high symptom burden. Five patients and three control participants refused to participate prior to inclusion. Further two additional patients dropped out prior to the first assessment due to the necessity of inpatient treatment.

Patients with AN were recruited at the outpatient centre for eating disorders of the Department for Child and Adolescent Psychiatry at the University of Mainz, Germany. The sample patients were treated at the outpatient centre on average 5.75 months (*SD* = 7.02 months) before enrolment in the study. Most of the patients (*N* = 14) received inpatient treatment previous to the study. Control participants were invited in local church groups and schools in the region of Mainz. Table 1 lists the demographic and clinical characteristics of the study population. Adolescents with and without AN did not differ regarding age or education.

**Table 1:** Characteristics of the study population

	Patients (*N* = 20)		Controls (*N* = 20)	
	*M*	*SD*	*M*	*SD*
Age (years)	16.0	1.55	15.9	1.95
BMI at first diagnostic assessment	16.5^a^	0.9^a^	--	--
Months enrolled in outpatient treatment	5.75	7.02	--	--
	*N*	*%*	*N*	*%*
*Education*				
A-level grammar school	15	75	14	70
Vocational school	0	0	1	5
Comprehensive school	1	5	3	15
Other (e.g. internship)	4	20	2	10
Restrictive AN-subtype	17	85		
Purging AN-subtype	3	15		
Previous inpatient treatment	14	70		
*Comorbidity*				
One additional disorder	6	30		
Two additional disorders	4	20		
*Comorbidity by disorder*				
Major Depression	9	45		
Bipolar Disorder	1	5		
OCD	2	10		
PTSD	1	5		
Adjustment disorder	1	5		

^a^ *N* = 19 as one patient refused weight measurement during diagnostic assessment

The extensive study protocol [36] was approved by the ethics committee of the regional medical association in Mainz, Germany, under the reference number 837.177.13 and registered at the German register of clinical trials (DRKS) under the register number DRKS00005228. The study was conducted according to the Helsinki Declaration. Informed consent was given by each participant and in the case of minors, their legal guardian.

In the current study, participants were administered a battery of self-report instruments. Patients with AN were additionally evaluated with a diagnostic interview as part of the standard procedure of the outpatient center. Afterwards, participants completed a questionnaire assessment on their smartphones during the EMA protocol. All measures are described below.

### Measures

Eating Disorder Examination adapted for Children [37]: With the German version of this semi-structured interview, the DSM-5 diagnoses of AN were established. The items of the original Eating Disorder Examination were adapted for administration to children and adolescents. The interview showed acceptable retest reliability over 7.5 months for most of the items in a community sample and excellent inter-rater reliability. It is thus considered a suitable instrument for confirming clinical diagnoses of eating disorders.

Symptom Check List (SCL-90-R) [35]: Prior to the EMA, all participants filled out the SCL-90-R. The SCL-90-R is an instrument for the assessment of general psychopathology and symptom burden. Internal consistencies were in between α = .74 and α = .97. The SCL-90-R has shown a test-retest reliability of *r* ≥ .69. Although the validity of the subscales of the SCL-90-R is discussed intensely, the global severity index showed a high validity regarding symptom burden. In this study, a cut-off value of 63 in the global severity index was used as an exclusion criterion for control participants with high psychological distress, as this value is considered to differentiate cases and controls [35].

EMA questionnaire: During the two-day momentary assessment all participants were asked repeatedly to fill out a short questionnaire, of which only two items were analysed in this study. To assess aversive tension, participants were asked directly how intense their aversive tension was at the present time from 0 to 100 in accordance with the German version of the DBT manual for adolescents [38] (“On a scale from 0—not present to 100—extremely intense, at this time, how intense is your emotional tension?”). In previous studies with patients with BPD, the assessment of aversive tension with EMA showed good validity and reliability both in the patient as in the control group [11, 12]. Prior to the assessment, a definition of aversive tension was given to the participants by stating that aversive tension is “a highly unpleasant emotional state accompanied by high arousal, which could prevent one from calming oneself during high levels of aversive tension.” However, to increase the reliability of the assessment and the understanding of the concept of aversive tension, the tension curve dividing aversive tension into three parts (low tension, 0–30; moderate tension, 30–70; and intolerable tension, 70–100) as provided in the DBT manual for adolescents [38] and DBT skills training manual [39] was explained to the participants. Additionally, all participants were given two examples of situations in which an aversive tension of 30 and 50 could be perceived.

Possible moderator events were assessed with an open-ended question asking what the participants had done immediately before answering the questionnaire. In case of simultaneous activities, participants were advised to fill in the most important event only. Importance was explained to the participants as the most time consuming event in the past hour or the event which in their subjective perception was mostly related to their current state of aversive tension. Prior to data analysis of events, all entered events were classified in four pre-specified categories: ‘food-intake-related’ , ‘sport-related’ , ‘school-related’ and ‘other’. Two raters categorized the events. If disagreement occurred, a third rater decided for one of the two proposed categories. Inter-rater-reliability of the two raters showed substantial agreement (Cohen’s Kappa, *κ* = .797, *p* < .001). As the study is part of a larger EMA trial, the participants answered two additional questions at every assessment time point, regarding how sure they were about their momentary emotions and which one they experienced at that moment. These two items were not analysed in this study.

### Procedure of the ecological momentary assessment

After enrolment and the baseline assessment, the EMA application Epicollect [40] was installed on the participants’ smartphone devices. Medical center devices were distributed to those without Android® smartphones. During initial assessment, all participants were trained on using the smartphone software and were given information on the aim of the study, aversive tension, and how to deal with any problems during participation. Two consecutive weekdays during the school week were chosen for participation, as weekends might differ regarding the perceived amount of aversive tension. This is in accordance with a study of adults with AN, which found different tension levels on weekends when compared to weekdays [41]. Previous studies found that 48h provide sufficient time for an hourly assessment of aversive tension [11, 42]. In line with Engel et al. [14], participants were advised to delay their entries whenever they felt unable to respond immediately, especially if the smartphone use was restricted during school times.

For this EMA study, a fixed signal-contingent schedule approach was used as described by Wheeler and Reis [43]. Fixed signal-contingent assessments require participants to respond to a signal at previously defined time points. In this study, participants received a short message to their personal smartphone at every full hour for two days, except during individually arranged sleeping times. To assess possible delays in responding, time stamps of data entries were saved. The data was copied to the study server through a local wireless network at a follow-up meeting. Patients were given the opportunity to share the data with their outpatient therapist.

### Data analyses

In the current analysis, only aversive tension and reported events were analysed. Data was analysed using SPSS 23 (IBM SPSS Statistics for Windows, Version 23.0, IBM, 2014), mixed model analyses were conducted with SAS software (SAS 9.4 University Edition for Windows, SAS Institute Inc., Cary, NC, USA, 2013).

Participants reported their perceived aversive tension hourly on two consecutive weekdays. Due to the naturalistic setting of the ambulatory monitoring, the participants differed in the number of their responses as they arranged individual sleeping times and missing entries occurred. To compute a mean value of aversive tension, values of aversive tension were aggregated personwise. T-tests were conducted to compare personal mean and maximum values of the patient and control group. To account for multiple testing, Bonferroni-Holm procedure was applied [44].

The data was analysed using a hierarchical linear model, which is recommended for analysing data obtained in momentary assessment [45, 46]. In line with the aims of the study, the following simplified general equation with fixed and random factors was formed:

$$ {y}_{ti}={\beta}_0+{\beta}_1TIM{E}_{ti}+{\beta}_2 GROU{P}_i+{\displaystyle \sum_j}{\beta}_j{X}_{tij}+{\displaystyle \sum_k}{\beta}_k{X}_{tik}\times GROU{P}_i+{r}_{0i}+{r}_{1i}TIM{E}_{ti}+{e}_{ti} $$

In this equation, Y_ti_ is the intensity of aversive tension of the person i at time point t, and β represents the fixed effects of time, group, events, and interaction effects of events and group. Accounting for residual variance on personal and time level, fixed and random effects of intercept and time were included. These factors are assumed to be independent and normally distributed with expectancies of zero. The specific variances are Var(*e*_*ti*_), Var(*r*_0*i*_), Var(*r*_1*i*_), and covariance of intercept and time Cov(*r*_0*i*_, *r*_1*i*_). Because of an assumed auto-correlation of aversive tension at adjacent measurement time points and the highly unequal intervals between those time points, a spatial power structure for autoregressive errors was assumed. The event categories were included as dummy variables coding 1 if the event was present and 0 if absent. The parameters of the model were estimated using the restricted maximum likelihood method (REML), as recommended if sample sizes are low.

To analyse the increase and decrease of aversive tension after a specific event, generalized logit mixed models were computed. Probabilities for effects on an increase or decrease of aversive tension were calculated. Meaningful changes of aversive tension were defined as an increase or decrease of at least 10 % within 90 min after the assessment of aversive tension as patients could delay their data entries. Increases and decreases are coded 1 if meaningful and 0 if little or no change. Fixed and random effects of intercept, time, and intercept-time-covariance were included. Due to the limited number of observations, reported events (i.e. food intake, school, sports) were included as fixed effects only.

## Results

### EMA measurements

The dataset consisted of 40 participants entering a total of 1030 completed observations. Patients and control participants did not differ significantly regarding the number of completed mobile questionnaires (Patients: *M* = 25.3, *SD* = 4.01, Controls: *M* = 26.2, *SD* = 5.91; *t*(38) = −.56, *p* = .576) and their compliance to the assessment (Patients: *M* = .79, *SD* = .12, Controls: *M* = .82, *SD* = .17; *t*(38) = .72, *p* = .476). Compliance was defined as the ratio of completed questionnaires to planned data entry time points. A medical center device was used by 19 participants (12 controls, 7 patients). There was no significant difference between the groups regarding the distribution of medical center devices (Fisher’s exact test *p*=.205). Table 2 provides the number of events experienced in the patient and control group. There were more food intake-related and fewer school- and sport-related events reported in the patient than in the control group. Adolescents with AN reported 354 observations with a subsequent observation within 90 min of which 100 (28.25 %) were increased and 97 (27.40 %) decreased in aversive tension by at least 10 %.

**Table 2:** Frequencies of the reported events of adolescents with and without AN

	Adolescents with AN	Healthy adolescents	Total
Food intake-related events	114	60	174
School-related events	95	124	219
Sports-related events	13	28	41
Other events	283	310	593
Total	505	522	1027

Events were not equally distributed: *χ* ^2^(3) = 27.04, *p* <.001. In three observations events were not reported

### Primary Outcomes

When compared to the control group (*M* = 17.10, *SD* = 11.92), the patient group (*M* = 44.06, *SD* = 22.75) reported as expected significantly higher mean values of aversive tension on an adjusted alpha-level of *α* = .025 (*t*(28.71) = 4.69, *p* ≤ .001) corresponding with a large effect size of *d* = 1.48 [47].

Additionally, higher maximum values of aversive tension in the patient group where found, in line with the hypothesis. An average maximum level of aversive tension of *M* = 71.30 (*SD* = 28.92) in the patient group was compared to the average maximum level of *M* = 39.55 (*SD* = 27.49) in the control group showing significant difference at an adjusted alpha-level of *α* = .05 (*t*(38) = 3.56, *p* = .001), with a large effect size of *d* = 1.13.

### Multilevel analyses

**Fig. 1:**
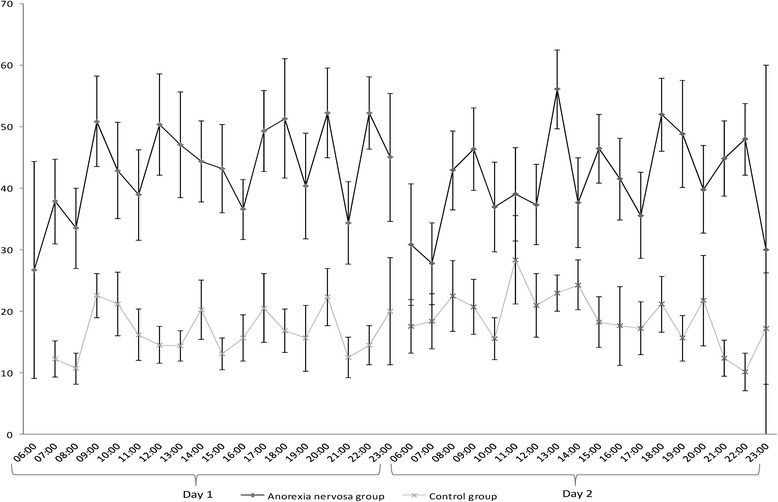
Time course of mean aversive tension over two days in the Anorexia nervosa and control group. Notes: Mean values of aversive tension at specific time points were only displayed if at least two participants of the specific group responded at the given time (+/− 30 min). Error bars represent standard error

Figure 1 illustrates the time courses of mean arsive tension of the AN and control group. To estimate effects of reported food intake, sports- and school-related events on momentary aversive tension, a multilevel model analyses was conducted. Fixed and random effect estimates of the model are provided in Table 3.

**Table 3:** Effect estimates of group, time, events and the interaction of group and events on the momentary experience of aversive tension: Fixed and random factor estimates

Fixed effects	*Estimate*	*SE*	*t* ^*a*^	*p* ^*b*^	Adjusted alpha-level^c^
Intercept	18.02	5.20	3.47	.001	α ≤ .0035
Time	0.01	0.06	0.25	.801	
Group	29.07	8.00	3.63	< .001	α ≤ .0035
Food Intake	0.54	1.58	0.34	.733	
Food Intake x Group	6.43	2.06	3.12	.004	α ≤ .005
School	1.41	1.46	0.97	.338	
School x Group	1.98	1.99	1.00	.326	
Sport	0.03	2.52	0.01	.992	
Sport x Group	−5.58	4.93	−1.13	.266	
Random effects	*Variance*	*SE*	*Wald Z*	*p* ^*b*^	
Intercept	312.32	80.13	3.90	< .001	α ≤ .0035
Time	0.07	0.03	2.57	.005	α ≤ .005
Intercept-time covariance	−0.48	1.12	−0.40	.689	
Autocorrelation	0.02	0.02	1.00	.320	
Residual	197.35	9.64	20.46	< .001	α ≤ .0035

Note: *N* = 40 persons, 1,027 observations. Group was coded controls = 0 and patients = 1, events 1 if present and 0 if absent immediately before responding. Therefore, group and event effects show additive effects for the patient group and if event was present

^a^We took a conservative approach to specify degrees of freedom, such that these were based on the number of participants (*N* = 40) and not on observations (*N* = 1,027). Degrees of freedom are therefore 39 for time, 38 for intercept and group, 36 for all additional effects

^b^All *p*-values are two-tailed expect in case of variances, where one-tailed *p*-values are used as variances are non-negative

^c^Alpha-error adjustment was conducted with the Bonferroni-Holm procedure

In line with the primary outcomes, diagnosis of AN was the largest influencing factor on aversive tension (main effect). This indicates that adolescents with AN experience more aversive tension in their daily-life than healthy adolescents. Furthermore, food intake influenced aversive tension significantly in patients with AN, but not in the control group, implying that previous food intake is associated with heightened aversive tension when compared to other situations in the life of adolescents with AN. School and sport-related events, however, did not influence aversive tension significantly, in the patient or in the control group. This is in contrast to our hypotheses, as we expected that school-related events would be associated with higher and sport-related events with lower levels of aversive tension at least in the patient group. Finally, random effects for intercept and time where found. The residual variance remained significant as well, indicating as expected that additional factors not investigated in the model may exist. No significant auto-correlation was found. Due to the REML method, no comparison of the model to the unconditioned model can be made, but a notable reduction of the −2 restricted log-likelihood was observed (unconditioned model [time and intercept only]: − 2*LL* = 8521.5; fully conditioned model: − 2*LL* = 8417.8).

Because of increases and decreases of subsequent levels of aversive tension in adolescents with AN, logit mixed model analyses were conducted. Table 4 provides the effect estimates of the increase and decrease models. In contrast to our hypothesis, we did not find any significant effect of time, school or sport-related events, on increased aversive tension of adolescents with AN within 90 min after the event. Neither sport- nor school-related events are related to a decrease of aversive tension. From a clinical point of view and for further investigations it is important to mention that reported food intake significantly reduced the number of subsequent increases of aversive tension in patients with AN. In addition, food intake was followed by a significant decrease of aversive tension within a period of 90 min afterwards.

**Table 4:** Effect estimates of events and time on subsequent increase or decrease of aversive tension in adolescents with AN

	Increases				Decreases			
Fixed effects	*Estimate*	*SE*	*t* ^*a*^	*p* ^*b*^	*Estimate*	*SE*	*t* ^*a*^	*p* ^*b*^
Intercept	−2.04	1.05	−1.95	.066	−0.90	1.20	−0.75	.461
Time	−0.02	0.01	−1.71	.105	−0.01	0.01	−0.94	.357
Food Intake	−0.86	0.33	−2.65	.017^*^	0.77	0.29	2.68	.016^*^
School	−0.63	0.35	−1.80	.090	−0.13	0.34	−0.37	.717
Sport	0.17	0.87	0.19	.849	−0.57	1.08	−0.53	.602
Random effects	*Variance*	*SE*			*Variance*	*SE*		
Intercept	0.69	0.68			0.21	0.46		
Time	0				< 0.01	< 0.01		
Covariance	< 0.01	0.01			< − 0.01	0.02		
Residual	0.94	0.07			0.95	0.08		

Note: *N* = 20 persons, 354 observations, increases and decreases were coded 1 if aversive tension changes of at least 10% and 0 if no or little change occurred within the following 90 min

*Significant effect at *α*=.05

^a^We took a conservative approach to specify degrees of freedom specified on number of subjects. Degrees of freedom are 19 each for intercept and time, 17 for all additional effects

^b^All *p*-values are two-tailed

## Discussion

The primary objective of this study was to examine aversive tension in adolescent patients with AN collected with personal smartphone devices in a natural environment. More specifically, we studied the effect of food intake, school and sport-related events on aversive tension in adolescents with AN, compared to healthy adolescents.

The main results are in line with our hypotheses that adolescents with AN experience higher mean and maximum levels of aversive tension when compared to healthy control participants. The findings indicate clearly the relevance of aversive tension as a psychological momentary factor in AN, as proposed in the transactional model of Haynos and Fruzzetti [7]. However, we did not assess whether restriction regulates aversive tension.

Additionally, we found that patients with AN experience not only a higher baseline level of aversive tension, but also more tension if a food-intake-related event was reported at the same time. These events did not have impact on the levels of aversive tension in the control group. However, events related to sport or school which might be perceived as stressful by adolescents did not have any impact on aversive tension neither in the control nor the patient group. This substantial interaction effect demonstrates the impact of regular food-intake-related events on the level of distress in patients with AN. Previous studies on momentary affective states in individuals with AN did not examine the current situation [14, 15]. Therefore, our study gives evidence that the momentary state of aversive tension is dependent on situational factors. As events were assessed with an open question, the finding that adolescents with AN reported substantially more food-intake-related events (e.g. meal times, being at a café) further underlines the significance of such events on the emotional state of adolescents with AN. In this study, the participants were obliged to report the most important event only. Hence, the effects of co-occurring events on aversive tension, possibly not noticed by the participants, were not assessed. The missing effect of other emotional events on aversive tension is presumably due to the rather extensive definition of school-related and the rare occurrence of sport-related events. Therefore, further studies should a priori define more limited categories, allow the co-occurrence of events and examine more than two days to detect further emotional events associated with aversive tension.

When examining changes of aversive tension within 90 min after a specific reported event, food intake was associated significantly with fewer increases and more decreases of aversive tension. Neither school-related nor sport-related events showed significant effects on change in aversive tension.

The latter findings are in contrast to our hypotheses and several explanations seem plausible. As we did not assess potentially disordered eating behavior, it is unknown if patients restricted their eating in these situations, used escape strategies or if maladaptive emotion regulation behavior (e.g. purging, excessive exercising after eating) occurred. However, it is reasonable to assume that adolescents with AN restrict their eating on most of the food-related events. If such behavior occurred, it may result in a decrease of subsequent aversive tension, as in line with the emotion regulation hypothesis proposed by Haynos and Fruzzetti [7]. The findings could also be indicating that aversive tension is triggered by the anticipation of events rather than during the actual situation itself. Subsequently, aversive tension should decrease once the situation has passed, whether or not the adolescent with AN has eaten. Terminating a meal situation could be associated with a great relief from emotional distress and therefore result in lower subsequent aversive tension. Restricted eating patterns or using escape strategies avoiding food intake resulting in decreased tension would serve as negative reinforcement and would therefore be an important factor of sustaining AN. An alternative explanation might be that food intake is associated with such high levels of aversive tension that a ceiling effect occurs and only further decreases were detectable. However, further studies on temporal effects with shorter measurement intervals are needed and should assess compensatory behavior as well.

The findings of this study have important implications for clinical practice. Aversive tension is a construct that significantly affects adolescents with AN in their daily life and should therefore be addressed in treatment. Hence, we suppose that patients with AN could benefit from distress tolerance skills as implemented in ‘traditional’ DBT. Especially skills for reducing aversive tension before or during meal times may improve compliance with feeding plans and weight management. These considerations are in contrast to recent DBT approaches for adults with AN. In the radically-open DBT proposal of Lynch et al. [28], a shortened distress tolerance skills section is promoted, stating that patients with AN are less likely to experience states of heightened aversive tension [28]. Therefore, future research should examine aversive tension in an adult AN population to justify whether or not DBT for AN should also focus on high states of aversive tension.

There are several strengths of the study that are worth noting. First, we assessed aversive tension in an adolescent sample. Recent EMA research in AN focussed mostly on adults [14, 15, 48, 49]. As emotion regulation differs strongly regarding age [22], it is unlikely that emotional processes in AN will remain stable from adolescence to adulthood. Second, assessing data in a momentary manner reduces substantially the retrospective recall bias of questionnaires. Therefore, later events could not influence the assessment of aversive tension of antecedent observations. Third, the EMA was conducted on modern smartphone devices which resulted in a high acceptance and excellent compliance with the method. To our knowledge, this was the first study allowing data assessment on personal smartphone devices of individuals with AN. However, only half of the participants used their personal smartphone because of the limitation to Android devices. Further research on real-life data should use an application available on at least Android and iOS® devices to further increase the rate of personal smartphones in the assessment. It is reasonable to assume that using personal devices increases data quality especially in adolescent study populations.

Notwithstanding, several limitations to the current study need to be addressed. First of all, the sample size of this study was rather small. Further research should examine aversive tension using larger samples, especially if additional moderator variables shall be examined. Further research regarding the influence of the severity or the subtype of AN could enhance the understanding of aversive tension in AN. Additionally, analysing the intrapersonal variability of aversive tension could provide further inside into understanding emotional dysregulation processes in this disorder. Some of the biases immanent to self-reported data (e.g. social desirability bias, exaggerations) could still affect EMA data despite their momentary character. Thus, measuring physiological data (e.g. heart rate variability, skin conductance level) at the same time could increase the reliability of the assessment of aversive tension. Additionally, we assessed aversive tension during weekdays. As previous findings showed significant differences in tension between weekdays and weekends [41], it is unclear if our findings generalize to weekends. Another limitation is that momentary disordered eating behavior was not assessed explicitly during the study, and was never reported voluntarily in the open question by the adolescents with AN. Accordingly, the data did not allow any analysis of the relation between aversive tension and, for example, restrictive or purging behavior. Previous EMA research on these behaviors did not investigate antecedent events [14, 15], and therefore, future research examining the path from emotional events to momentary negative affect and aversive tension to disordered eating is needed. Furthermore, adolescents with AN reported significantly more food-related events than the control group, indicating that they either have indeed more meal times (but with a smaller calorie intake) than adolescents without AN, or that food intake is of such importance for adolescents with AN that reporting seems more significant to them. Measuring food intake objectively (e.g. in an inpatient setting) could therefore provide further insight into the relation of food intake and aversive tension.

## Conclusions

Overall, the findings of this study provide support for the emotion dysregulation model of AN. Aversive tension seems relevant in AN and should be addressed in treatment. However, it remains unclear if aversive tension is limited to adolescent AN, as only adolescents were examined. The occurrence of events related to food intake is associated with higher levels of aversive tension. Therefore, teaching adolescents with AN how to reduce aversive tension could result in a better compliance during weight restoration. In addition to clear evidence underlining the model of Haynos and Fruzetti [7], the role of aversive tension in the path from potentially emotional events via cognition, emotion and behavior to disordered eating should be addressed further.

### Ethics approval and consent to participate

This study received a positive ethic approval from the ethics committee of the regional medical association in Mainz, Germany (reference number: 837.177.13). Permission to conduct the study was also granted by the relevant clinical director for the mental health service involved. All participants and their legal guardians were provided with a participant information sheet and provided their signed informed consent to participate in the study.

### Consent for publication

As part of ethics approval and consent, all participants also provided their consent for their de-identified data to be published.

### Availability of data and materials

As our questionnaire contained several open text fields asking for current activities, the raw data is not anonymous and might personally identify participants. Therefore, raw data will not be made publically available. The de-identified data can be made available upon request to the corresponding author.

## Abbreviations

AN: Anorexia nerovsa
BPD: Borderline personality disorder
DBT: Dialectical behavior therapy
DRKS: German register of clinical trials
EMA: Ecological momentary assessment
REML: Restricted maximum-likelihood
SCL-90-R: Symptom checklist 90-R
